# The Role of Hydrolysed Rice Formula in the Dietary Management of Infants with Cow’s Milk Allergy: A UK Healthcare Perspective

**DOI:** 10.3390/nu18081225

**Published:** 2026-04-14

**Authors:** Nick Makwana, Lauren Arpe, Aneta Ivanova, Helen Evans-Howells, Claire Trigg, Bahee Van de Bor, Joanne Walsh, Annette Weaver, Rachel Wood, Carina Venter, Yvan Vandenplas, Rosan Meyer

**Affiliations:** 1 Sandwell and West Birmingham Hospitals, Birmingham B71 4HJ, UK; aneta.ivanova@nhs.net; 2 Birmingham Medical School, University of Birmingham, Birmingham B15 2TT, UK; 3 Gastroenterology Department, Great Ormond Street Hospital, London WC1N 3JH, UK; lauren.arpe@gosh.nhs.uk; 4 Dr Helen Allergy, Ringwood BH24 1HP, UK; h.howells@nhs.net; 5 Royal Glamorgan Hospital, Ynysmaerdy, Pontyclun CF72 8XR, UK; claire.trigg@wales.nhs.uk; 6 Princess of Wales Hospital, Coity Road, Bridgend CF31 1RQ, UK; 7 UK Kids Nutrition, London W1G 9PF, UK; bahee@ukkidsnutrition.com; 8 Castle Partnership, Norwich NR3 1SE, UK; joanne.walsh@nhs.net; 9 Allergy UK, Crayford DA1 4SL, UK; info@allergyuk.org; 10 Manchester Children’s Hospital, Manchester M13 9WL, UK; rachel.wood@mft.nhs.uk; 11 Section of Allergy and Clinical Immunology, Children’s Hospital Colorado, University of Colorado, Aurora, CO 80045, USA; carina.venter@childrenscolorado.org; 12 KidZ Health Castle, UZ Brussel, Vrije Universiteit Brussel (VUB), 1090 Brussels, Belgium; yvan.vandenplas@uzbrussel.be; 13 Department Medicine, KU Leuven, 3000 Leuven, Belgium; info@rosan-paediatricdietitian.com

**Keywords:** cow’s milk allergy, hydrolysed formula, hydrolysed rice formula, infant allergy, infant feeding

## Abstract

Cow’s milk allergy (CMA) remains one of the most common food allergies in infancy, requiring the avoidance of cow’s milk and its derivatives. Breast milk is the best source of nutrition for infants. For those infants with CMA whose mothers are unable to breastfeed or choose not to, extensively hydrolysed formulas (eHFs) are widely recommended as first-line milk substitutes, whereas hydrolysed rice formulas (HRFs) are increasingly recognised as a viable alternative. This concept paper provides a healthcare professional (HCP) perspective on HRF, drawing on expert consensus from two meetings convened in 2025. Discussions noted the long history of safe and effective HRF use in Europe, its nutritional adequacy, and the evolving international guidelines supporting HRF as an alternative first-line option. A key meeting outcome was the development of a practical decision tree to help UK clinicians decide when HRF should be the preferred choice. Key considerations for its use in non-breastfed infants include the following: parental/caregiver stress related to persistent symptoms; ongoing symptoms despite multiple interventions; cultural and lifestyle choices; religious dietary requirements; and specialists’ recommendations. Secondary considerations highlighted by HCPs include the following: proven reactions whilst infants are breast-milk-fed together with parental request for formula; faltering growth; multiple symptoms; taste acceptance (older infants); and parental preference based on experience. The role of functional components, such as prebiotics and human milk oligosaccharides (HMOs), was noted in regard to the emerging evidence of benefits to the microbiome and immune development. The experts emphasised the importance of engaging HCPs across all levels of CMA care and addressing challenges in translating current guidance into treatment practice. It was concluded that, overall, HRF represents a nutritionally complete, plant-based alternative that has been shown to be well tolerated (taste, symptoms) in clinical studies. It can be used to broaden therapeutic options for infants with CMA in the UK who are not exclusively fed breast milk.

## 1. Introduction

The World Health Organization recommends exclusive breast milk feeding for the first 6 months as the gold standard for infant feeding, followed by continued breast milk feeding alongside complementary solid foods, ideally up to 2 years of age and beyond [1]. Cow’s milk allergy (CMA) is one of the most common food allergies in infants, although most children outgrow it by the age of 3–5 years [2,3]. The prevalence of food allergies appears to be rising globally [4,5,6] although recent evidence from the UK suggests that the rate of new food allergy cases may be stabilising, particularly in preschool children [7]. There is an increased awareness among parents of the symptoms of CMA, as well as an overall improved recognition of its prevalence by healthcare professionals (HCPs) [8,9]. CMA presents with diverse symptoms that can significantly affect both children and their caregivers [10]. Effective care requires the avoidance of cow’s milk while ensuring the use of appropriate nutritional substitutes to maintain healthy growth and development [11].

For infants with CMA, maternal exclusion of cow’s milk and its derivatives is only required in a small number of cases [1,12]. For mothers who cannot breastfeed or choose not to, extensively hydrolysed cow’s milk-based formulas (eHFs) or hydrolysed rice formulas (HRFs), where available, are the first-line options for infants with CMA, as endorsed by the European Society for Paediatric Gastroenterology, Hepatology and Nutrition (ESPGHAN) and World Allergy Organization (WAO) Diagnosis and Rationale for Action against Cow’s Milk Allergy (DRACMA) guidelines [13,14]. If symptoms do not improve with eHF or HRF, use of an amino acid formula (AAF) should be considered, because some infants may react to the residual peptides in the eHF, but it is also important in the diagnosis to eliminate other possible causes of symptoms [15]. An AAF as first-line treatment is generally reserved only for severe presentations, such as eosinophilic oesophagitis (EoE) [16].

Recent international guidelines acknowledge that an HRF or eHF is a suitable first-line treatment option for infants with CMA [13,14]. The Australasian Society of Clinical Immunology and Allergy (ASCIA) also suggests the first-line use of HRF for anaphylaxis and food protein-induced enterocolitis syndrome (FPIES) [17]. In contrast, older guidelines (e.g., British Society for Allergy and Clinical Immunology [BSACI] 2014) reflect the formula options available at that time and therefore do not include HRF [18].

This paper reports the outcomes of 2025 UK expert discussions from two meetings, from which a decision tree was developed to determine in which circumstances HRF should be the preferred option for infants with CMA in the UK. This decision tree is not intended as a replacement for current or future published official guidelines on CMA.

## 2. Background: UK Expert Meetings

Two expert meetings with UK healthcare professionals were convened in 2025. The first (15 May) was a face-to-face meeting with eight HCPs (six paediatric specialty dietitians, a paediatric allergy nurse consultant, and a paediatric gastroenterologist) and was followed by a virtual meeting (9 July) with six HCPs (an allergy/gastrointestinal (GI) specialist paediatric dietitian, a specialist allergy dietitian, a paediatric allergy nurse consultant, a paediatric allergist, and two general practitioners with a special interest in allergy). Both meetings were chaired by a paediatric dietitian (R.M.) specialising in food hypersensitivity. The primary aim of the meetings was to develop a decision tree to support HCPs in selecting a suitable formula, from the multiple options available (including HRF in the UK), for non-breastfed infants with CMA (Box 1).

Box 1 **Meeting objectives/goals.**

1. To discuss current clinical practice and UK guidelines in the context of international guidelines and decision-making around the nutritional management of CMA and other circumstances in which HRF is clinically indicated.
2. To discuss the evidence-based science supporting the clinical benefits of using HRF in the management of CMA.
3. To explore HCPs’ perceptions, barriers, and awareness of current CMA guidelines, with reference to clinical practice in the use of HRF.
4. To explore the increased number of choices of infant food(s) for special medical purposes categories that are now available for infants with CMA and the potential benefits of HRF vs. the current standard of care.
5. To develop an HRF decision tree for clinical care to support clinicians during the diagnosis and dietary management of CMA in the UK.
6. To identify any additional needs, from an HCP perspective, to ensure the HRF decision tree is comprehensive and reflects clinical practice in the UK.

CMA, cow’s milk allergy; HCP, healthcare professional; HRF, hydrolysed rice formula

## 3. Summary of Expert Meetings

The first meeting comprised five sections: (1) history of HRF in the EU and an update on international guidelines for CMA (presented by Y.V.); (2) CMA assessment and diagnosis (presented by C.V.); (3) current dietary management of CMA in the UK (presented by R.M.); (4) UK prescribing behaviours (facilitated discussion session); and (5) workshop session dedicated to developing the clinical decision tree.

The second meeting comprised two sections: (1) an overview of current CMA management guidelines and a narrative review of HRF and current evidence (presented by RM); and (2) a workshop session focused on reviewing and refining the HRF decision tree developed initially in the first meeting.

## 4. Development of HRF Decision Tree

**Figure 1:**
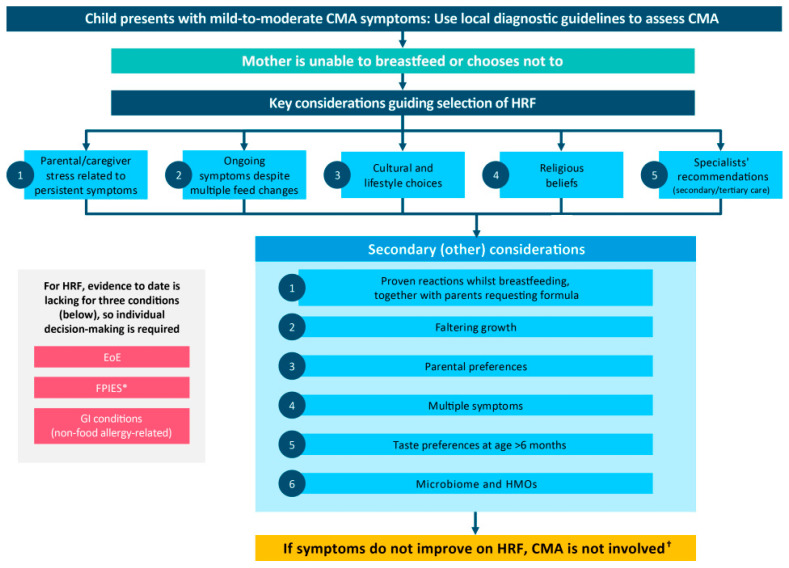
Decision tree to determine when HRF is the preferred formula for infants with CMA. * DRACMA guidelines state, “In infants with milk- or rice-related FPIES, or in whom tolerance of rice is unknown, it is more beneficial not to use HRF until more evidence is available” [13]. The expert panel noted this reflects limited data and the reported frequency of rice-related FPIES. ^†^ As advised in the most recently available (2024) ESPGHAN algorithm at the time of publication [14]. Abbreviations: CMA, cow’s milk allergy; EoE, eosinophilic esophagitis; FPIES, food protein-induced enterocolitis syndrome; GI, gastrointestinal; HMO, human milk oligosaccharide; HRF, hydrolysed rice formula.

The refined version of the decision tree, developed at the second meeting, was circulated offline to all participants and finalised (Figure 1). The decision tree is not intended as a diagnostic or treatment algorithm but rather as a tool to support clinicians when choosing a specialist formula for infants who are not exclusively breastfed. When children present with symptoms suggestive of CMA, local diagnostic guidelines should be followed to confirm the diagnosis. The decision tree should be interpreted alongside this accompanying article, which outlines its development.

Breastfeeding should always be encouraged and supported as the preferred source of nutrition [19,20]. If the mother is unable to breastfeed or chooses not to, or if breast milk is insufficient to meet the infant’s needs, formula will be required for infants with CMA, and eHFs and HRFs (where available) are recommended as first-line options [13,14]. Although HRFs were not historically included in all recommendations, the recent literature and guidelines have increasingly recognised HRF as a valid first-line alternative to eHF. Importantly, HRFs have been used in Europe for more than 20 years without any reported adverse effects on infant health [21].

The five key considerations, discussed during the expert meetings, for determining when an HRF is the preferred choice for an infant with suspected or confirmed CMA are listed and summarised in Table 1.

Secondary (other) considerations that were discussed by experts are listed and summarised in Table 2. It should also be noted that HRF also has a low osmolality, which may be better tolerated in children with reflux and gastrointestinal problems, but there is currently no evidence to substantiate this. Additional factors such as the impact on the gut microbiome and the presence of HMOs may also be relevant (Table 2).

If both eHF and HRF are ineffective (poor relief of symptoms and nutritional inadequacy in terms of growth support) or well tolerated, and CMA is still suspected, guidelines suggest that an AAF should be considered as a second-line option. However, ESPGHAN guidance (2024) suggests that if symptoms do not improve on HRF and cow’s milk is appropriately removed from an infant on complementary foods, then CMA is not involved [14]. For severe presentations, such as EoE, FPIES, and other non-food allergic GI conditions (i.e. short gut syndrome), HRF is not presently recommended in guidelines in the current absence of supporting evidence.

## 5. Conclusions

The management of CMA in infancy requires access to safe, nutritionally complete, and well-tolerated formula options when breast milk feeding is not possible or chosen. HRFs have demonstrated safety, tolerance, and nutritional adequacy over more than two decades of use in Europe and are increasingly recognised as a viable first-line alternative to eHF. New guidelines recommend either HRF or eHF as first-line feeds for infants with CMA who are not fed breast milk. There is, however, no evidence-based guideline on when an HRF may be preferred. The expert group set out to develop a practical decision tree to support HCPs in determining when an HRF is the preferred option for the dietary management of non-breastfed infants with CMA, reflecting the evolving evidence base and alignment with international practice. The practical decision tree developed by the expert group, which incorporates both clinical and psychosocial considerations, as well as those acknowledging individual family preferences, offers a structured framework to assist HCPs when making such decisions.

Broader awareness, education, and equitable access to HRF are essential to ensure optimal care for infants with CMA in the UK. Future research should continue to investigate long-term outcomes, including the impact of HRF on growth, immunity, and the gut microbiome, to further explore and strengthen the evidence supporting HRF use in non-breastfed infants. Overall, HRF represents an effective, well-tolerated, plant-based alternative that broadens the therapeutic options available to clinicians and supports a more personalised, family-centred approach to CMA management.

## Figures and Tables

**Table 1:** Key considerations guiding the selection of HRF.

Key Considerations	Notes
1. Parental/caregiver stress related to persistent symptoms	Parents or caregivers may be overwhelmed by persistent symptoms of CMA. Ongoing symptoms not only impact the quality of life of the infant and family but may also lead to feeding difficulties and affect growth and nutritional status [22]. After multiple unsuccessful formula trials, parents might seek a formula that excludes cow’s milk protein entirely, such as an HRF.
2. Ongoing symptoms despite multiple feed changes	If an infant continues to experience symptoms such as GI discomfort (diarrhoea, blood in stool, abdominal pain) or skin issues, where multiple feed changes (standard infant formulas, comfort formulas, eHF for 2–4 weeks) have occurred, an HRF might be considered because it eliminates residual cow’s milk protein entirely, potentially leading to better symptom resolution.
3. Cultural and lifestyle choices	HCPs are increasingly encountering families whose cultural or lifestyle values prohibit or discourage the consumption of cow’s milk formula. Families adhering to an animal protein-free lifestyle may prefer HRF because of its plant-based * origin, which aligns with their dietary values and ethical considerations [13,23].
4. Religious beliefs	Religious beliefs influence the nutritional choices parents make for their children [24]. HRFs are suitable for families with specific religious dietary requirements. Certification is product-specific, so it is essential to check with the infant formula manufacturer before use [21,25].
5. Specialists’ recommendations	HCPs across the spectrum of primary to tertiary care should be engaged in the decision-making process when introducing a new formula; however, significant challenges remain in translating guidance into clinical practice [26]. Managing parental expectations can be difficult for HCPs and may influence decisions around formula switching [26].

* HRFs do not contain lactose but are not always 100% vegan, because some may contain trace amounts of a biosynthesised ingredient derived from lactose (e.g., HMO). Such HMOs are structurally equivalent to those present in human milk and are produced via a fermentation process similar to that used for some vitamins (e.g., vitamin B2). Abbreviations: CMA, cow’s milk allergy; eHF, extensively hydrolysed formula; GI, gastrointestinal; HCP, healthcare professional; HMO, human milk oligosaccharide; HRF, hydrolysed rice formula.

**Table 2:** Secondary considerations guiding the selection of HRF.

Secondary Considerations	Notes
1. Proven symptoms whilst breastfed and parental request for formula	Infants who are no longer breastfed or who require top-up formula—and in whom there is a proven reaction, through elimination and reintroduction, to low levels of beta lactoglobulin in breast milk * due to maternal consumption of cow’s milk—may benefit from HRF, which is entirely free of cow’s milk protein without traces of beta lactoglobulin.
2. Faltering growth	If an infant meets the NICE criteria for faltering growth, and this is considered possibly related to an intolerance of the residual peptides in eHF, an HRF may be trialled, with growth monitoring at intervals recommended by NICE NG75. ^†^ It should be noted that, for non-breastfed infants with CMA and faltering growth, most guidelines suggest an AAF as a first-line choice.
3. Parental preferences	Parents with prior experience managing CMA may have unique, valuable insights into what was effective for their other children. If HRF was effective previously, they may prefer to use it again.
4. Multiple symptoms	In infants with complex presentations involving multiple organs (i.e., skin, gastrointestinal, and respiratory manifestations), HRF may be considered when exclusion of cow’s milk protein is clinically warranted.
5. Taste preferences in infants older than 6 months	Older infants may reject the taste of eHF or AAF. Due to the hydrolysis of cow’s milk proteins and the presence of free amino acids, these formulas can exhibit distinct smell, texture, taste, and aftertaste profiles that may have a potential long-term influence on patient preferences [12,20,24]. HRF may be offered as a different taste option, which may be better accepted by some infants.
6. The microbiome and HMOs	Infant formulas may differ in the inclusion of cow’s milk protein-free lactose, prebiotics, HMOs, probiotics, and synbiotics, which are all designed to support the developing microbiome in a manner more closely resembling that of breastfed infants and to promote immune tolerance [14,22]. Emerging data suggest that HRF supplemented with HMOs or prebiotics may influence the microbiome; longer-term studies are needed to assess the clinical relevance of such findings [27].

* Reactivity through breast milk is uncommon, and care should be taken to avoid unwarranted maternal elimination diets. ^†^ E.g. monitoring in infants: weekly if 1–6 months, fortnightly if 6–12 months, monthly if >1 year; length/height recorded ≤ every 3 months [28]. Abbreviations: AAF, amino acid formula; CMA, cow’s milk allergy; eHF, extensively hydrolysed cow’s milk-based formulas; HMO, human milk oligosaccharide; HRF, hydrolysed rice formula; NICE, National Institute for Health and Care Excellence.

## Data Availability

No new data were created or analysed in this study. Data sharing is not applicable to this article.
